# Age does not improve the predictive ability of the Hospital Frailty Risk Score for length of stay

**DOI:** 10.1371/journal.pone.0330930

**Published:** 2025-09-09

**Authors:** Huda Kutrani, Jim Briggs, David Prytherch, Claire Spice

**Affiliations:** 1 Centre for Healthcare Modelling and Informatics, University of Portsmouth, Portsmouth, United Kingdom; 2 Faculty of Public Health, University of Benghazi, Benghazi, Libya; 3 Queen Alexandra Hospital, Portsmouth University Hospitals NHS Trust, Portsmouth, United Kingdom; Universita degli Studi di Napoli Federico II, ITALY

## Abstract

**Background:**

The Hospital Frailty Risk Score (HFRS) has been widely used to identify patients at high risk of poor outcomes and to predict poor outcomes for older people. Although poor health outcomes are associated more with frailty than age, HFRS has been validated only for older people. This study aimed to explore for the first time whether age influences the predictive power of Hospital Frailty Risk Score to predict a long length of stay.

**Methods:**

A retrospective cohort study analysing data collected at Queen Alexandra Hospital in Portsmouth, UK, between January 1, 2010 and December 31, 2019. Data included people aged ≥16 years. We assessed the correlation between the HFRS and age using Pearson’s correlation coefficient. We used logistic regression models to develop prediction models (HFRS alone and HFRS +age) for nine periods of length of stay, in nine age groups data to assess association and influences age on Hospital Frailty Risk Score.

**Results:**

The correlation between Hospital Frailty Risk Score and age was weak in eight age groups, correlation coefficient ranged from 0.01 to 0.17. In each age groups, the proportion of the intermediate and high risk of frailty increased with a longer length of stay. Adjusted models (HFRS+age) did not show a better discriminative power compared with HFRS alone in all age groups (AUROCs ranging from 0.772 to 0.932). The odds ratio values from HFRS alone models were higher than adjusted models.

**Conclusions:**

This study concluded that in this patients’ population, age does not improve the power of Hospital Frailty Risk Score to predict long length of stay.

## Introduction

Frailty is a medical syndrome associated with an increased risk of poor health outcomes, including longer length of stay (LOS) [1–4]. Early identification of patients at risk of frailty reduces poor health outcomes, improves the quality of healthcare services and patient care, and reduces the costs of medical services [3,5–8]. and it is increasingly important to identify people at risk of frailty and its associated consequences.

The key conceptual constructs for frailty include the frailty phenotype, the accumulation of deficits, and multi-dimensional frailty model. There are many different instruments, taking a variety of approaches stemming from these concepts, to identify and quantify frailty including those that require direct assessment, such as deficit accumulation index [9] (e.g., multidimensional frailty score (m-Fi) [10], and fr-AGILE [11]), and functional judgement scales (e.g., Clinical Frailty Scale [12]). The Hospital Frailty Risk Score [13], based on electronic information and not requiring direct assessment by a clinician, was developed and validated in secondary care within the UK National Health Service [13]. It was calculated based on 109 ICD-10 diagnosis codes related to frailty, to identify patients at high risk of frailty and predict poor outcomes for older people [13]. And it offers a key advantage in that it can be integrated into most hospital information systems rapidly, cost-effectively, and with minimal impact on clinical staff workload [13–16]. As a result, HFRS has been widely used and validated to identify patients at high risk of poor outcomes [5,6,15–29].

Many studies using the HFRS confirmed that it assisted in identifying patients at risk of frailty and was associated with adverse outcomes but, like the original study, focused on older patients (>=75 years) [5,6,17,20–22]. Some studies looked across all ages but focused on specific conditions such as heart disease, finding that HFRS remained a significant predictor of adverse outcomes in those patient groups [23–27]. A recent study reported use of HFRS in predicting longer hospital length of stay across all ages [30].

Frail and sicker patients have a relatively longer hospital stay than others, and use more hospital resources; therefore, the hospital length of stay (LOS) is a potentially important indicator for measuring morbidity and the cost of medical care [31,32]. Predicting patients at risk of longer LOS could help focus on this group of patients early in their stay, to potentially reduce their length of stay and hospital resource use [31,32].

Frailty tends to increase with age [1–4,33], but poor health outcomes are associated more with frailty than age [2,33–37]. Previous studies reported that frailty was a strong predictor of mortality in all adult ages, and frail people had more health resource use [33,34,37,38]. Casazza et al (2020) found that frailty, not age, was an independent predictor for longer LOS across all adult ages [39]. Also, Gordon et al (2023) stated that frailty increased the risk of long length of stay in younger adults [4]. Dicpinigaitis et al. (2021) reported that in all adult ages, increasing frailty was independently associated with longer LOS, while increasing age was not [40]. While Joseph et al. (2014) found no association between age and the Frailty Index (FI), and frailty was superior to age in predicting poor outcomes [36]. Coleman et al. (2020) reported that frailty was more strongly associated with morbidity than older age in patients undergoing proctectomy [35]. These indicate that age does not necessarily explain patients’ health status.

Frailty increases with increasing age, but not all older adults develop frailty, and some younger adults do [1,2,4,33,41,42]. It has been postulated that the predictive validity of frailty tools may be due to the higher prevalence of frailty in older people [43]. This study aimed to explore for the first time whether adjusting the HFRS for age, or adding age as a variable, changed the ability of the Hospital Frailty Risk Score to predict a long length of stay.

## Methods

### Study design and participants

This study follows similar methods to those of Kutrani et al. (2025), which we summarise here for convenience [30]. This is a retrospective cohort study of adult patients aged 16 years and older, who were admitted from 1st January 2010–31st December 2019 (1,314,279 admissions) to a large acute hospital (Queen Alexandra Hospital) in Portsmouth, UK.

**Fig 1:**
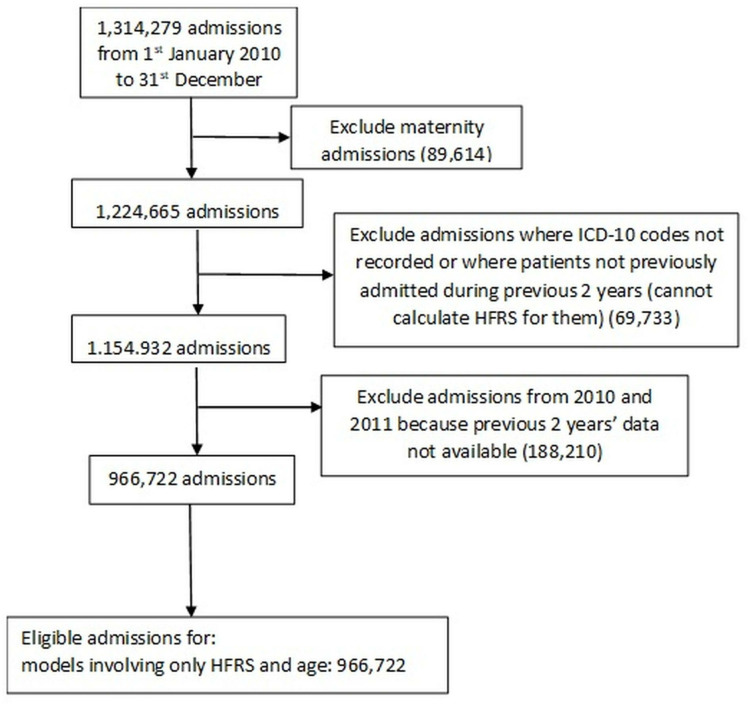
Flowchart of data exclusions.

Optimally, HFRS relies on having 2 previous years’ data [17]. Our source dataset contains admissions from 1st January 2010–31st December 2019, but we constrained our analysis to the period from 1st January 2012–31st December 2019 so that we could calculate HFRS optimally. We excluded certain categories of admissions such as maternity cases (see Fig 1).

For sub-analyses, we generated 8 age-band sub-datasets (16–24, 25–34, 35–44, and so on up to 85 and over) to explore whether age influences the predictive power of HFRS for the nine periods of LOS.

### HFRS calculation

We used the methodology described by the original study [13]. We calculated HFRS for each individual based on points associated with 109 ICD-10 diagnosis codes recorded in patients’ hospital admission records. The score is a weighted aggregate of the points for each code present in the index admission and in all earlier admissions during the previous two years. In addition to the score, the HFRS was categorised as low frailty risk (<5), intermediate frailty risk (5–15), and high frailty risk (>15) [13].

### Outcomes and variables

Thresholds for length of stay, rather than LOS as a continuous variable, might be more useful in the clinical and operational hospital context to focus efforts on particular groups. Examples include shorter stays (often less than 3 days) and longer stays. There is no international standard definition of a long stay. For example, the NHS defines a long stay as LOS > 21 days [44]. Several studies focus on LOS > 10 days [13,17,19,22,25], which may not be clinically relevant enough to capture very long hospital stays associated with patient complexity. We explore different periods of LOS to provide finer-grained analysis.

The association and predictive ability on LOS of the HFRS tool was evaluated for nine periods as outcomes of the study. These prediction periods were:

1. LOS > 3 days,
2. LOS > 7 days,
3. LOS > 10 days,
4. LOS > 14 days,
5. LOS > 21 days,
6. LOS > 30 days,
7. LOS > 45 days,
8. LOS > 60 days and
9. LOS > 90 days.

Variables were HFRS and age.

### Statistical analysis

Characteristics of each age group were summarised with frequency and percentage. We assessed the correlation of the continuous HFRS score and continuous age using Pearson’s correlation coefficient. Correlation below 0.40 was considered weak; values ranging from 0.40–0.69 was considered moderate; 0.70 or above was considered strong [45].

We developed a pair of logistic regression models for each LOS period, for each of eight age groups plus the “all ages” group. Each pair consisted of a model based on HFRS score alone and one combining HFRS score with continuous age.

Associations between HFRS and age with each LOS in the “all ages” group were evaluated by Odds Ratio. Discrimination and performance of models were assessed using AUROC (also known as the c-statistic). A value of AUROC below 0.60 indicates no discrimination; values ranging from 0.60–0.69 indicate poor discrimination, values of 0.70–0.79 indicate fair discrimination, values from 0.80–0.89 indicate good discrimination, and values of 0.90 and above indicate excellent discrimination [46]. We compared the AUROC value of the HFRS-alone models with HFRS combined with age models for each period of LOS in each age group’s data. Thus, a higher AUROC indicated better performance of the model. Paired t-test and Wilcoxon signed-rank test were used to check whether the difference in AUROCs values between HFRS alone models and HFRS + age models are statistically significant (does adding age significantly improve predictive performance of HFRS). P values <0.05 were considered statistically significant. Data manipulation and logistic regression modelling were performed using RStudio version 4.2.1.

### Ethics

The study included patients aged 16 years and older who had not registered for the UK national data opt-out to stop their data being used for research. We obtained pseudonymised data from the Portsmouth CORE-D routine care data repository on 1st November 2022. The researchers had no information that could identify individual patients during or after accessing data.

The dataset used in this study is covered by existing ethical approval granted by an NHS Research Ethics Committee in April 2021. REC reference is 21/SC/0080. IRAS project ID is 281193. The data is available from Portsmouth Hospital University NHS Trust under a data sharing agreement.

## Results

After exclusions, the data included 966,722 patients aged 16 years and older who were admitted to Queen Alexandra Hospital in Portsmouth, UK. We generated eight subsets according to age group to explore whether age is an influence on the predictive power of Hospital Frailty Risk Score (HFRS) to predict a long LOS.

Table 1 shows the association and relationship between HFRS and age. Although the majority of patients aged 84 and younger had low frailty risk, the proportion of admissions with HFRS greater than 0 was 60.2% (582,267 of 966,722 admissions) across “all ages”. Of patients aged 75 years and older, 72.4% (209,055 of 288,842 admissions) had HFRS greater than 0, compared with 55.1% (373,212 of 677,880 admissions) of patients younger than 75 years. Across eight age groups, the correlation coefficient between HFRS and age was weak, ranging from 0.01 to 0.17. In the “all ages” group, the correlation coefficient was slightly better (0.29) but still weak.

**Table 1:** Relation between HFRS and age.

Age groups data	No. of admissions	Number of admissions with HFRS greater than 0 N (%)	Hospital Frailty Risk Score category			Correlation between HFRS and age
			Low risk (<5)	Intermediate risk (5-15)	High risk (>15)
all ages	966,722	582,267 (60.2%)	747,265 (77.3%)	150,365 (15.6%)	69,092 (7.1%)	0.29
16-24 years	43,109	21,807 (50.6%)	39,683 (92.1%)	3,073 (7.1%)	353 (0.8%)	0.06
25-34 years	72,514	36,784 (50.7%)	65,598 (91.4%)	6,133 (8.5%)	783 (1.1%)	0.01
35-44 years	75,919	40,705 (53.6%)	67,663 (89.1%)	7,185 (9.5%)	1,071 (1.4%)	0.01
45-54 years	120,971	66,936 (55.3%)	104,465 (86.4%)	13,638 (11.3%)	2,868 (2.4%)	0.02
55-64 years	160,940	87,605 (54.4%)	136,394 (84.8%)	19,379 (12.0%)	5,167 (3.2%)	0.06
65-74 years	204,427	119,375 (58.4%)	162,442 (79.5%)	31,861 (15.5%)	10,124 (5.0%)	0.07
75-84 years	182,234	123,129 (67.6%)	123,320 (67.6%)	38,185 (21.0%)	20,729 (11.4%)	0.12
85 years &more	106,608	85,926 (80.6%)	47,700 (44.7%)	30,911 (29.0%)	27,997 (26.3%)	0.17

The characteristics of the study population by age groups are presented in S1 Table. The risk of in-hospital mortality increased with age. Most patients aged less than 45 or over 84 years were admitted as non-elective cases. From the top ten admission specialities, general medicine, gynaecology, and accident & emergency were the most common specialities in patients aged less than 45 years. For patients aged 45–74 years, general medicine, gastroenterology, and medical oncology were the most common specialities. For older patients, general medicine, gastroenterology, and accident & emergency were most common (S1 Table and S1 Fig).

**Fig 2:**
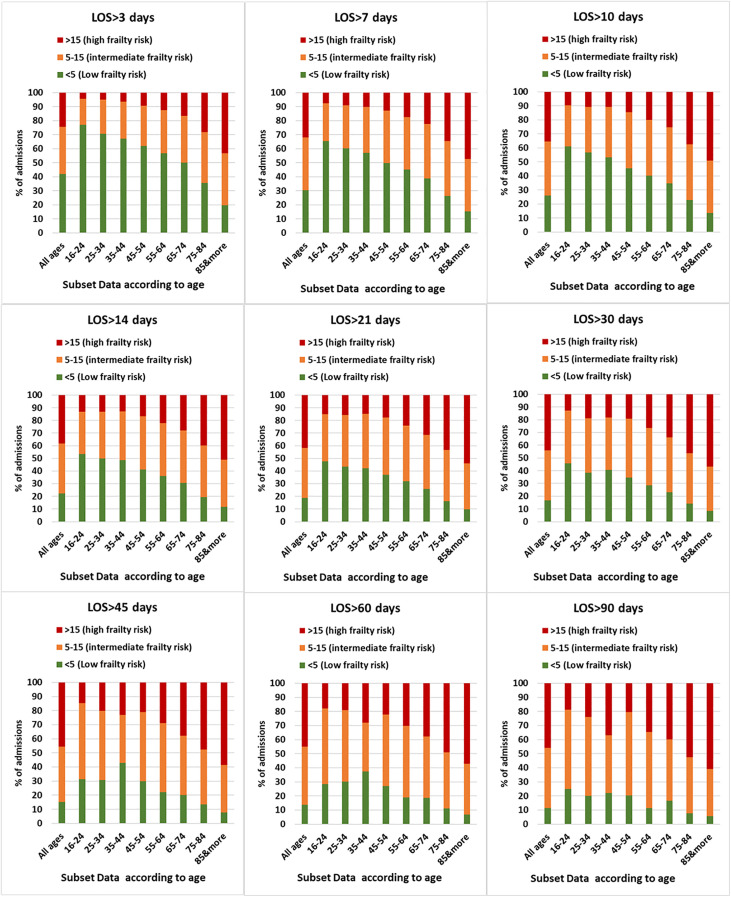
Association between Hospital Frailty Risk Score and age groups for 9 periods of LOS.

The association between HFRS and age groups for nine prediction periods of length of stay is shown in Fig 2 and the S2 Table. In general, an increase in intermediate or high frailty risk was associated with a longer length of stay in the hospital for all age groups. At least 52% of patients with intermediate or high frailty risk in each age group had length of stay in excess of 21 days. While at least 50% of patients aged 45 years and above with intermediate or high frailty risk had lengths of stay in excess of 7 days.

Moreover, in each age group, the proportion of intermediate and high risk of frailty increased with length of stay. Thus, more frailty means longer stays, for all age groups.

**Fig 3:**
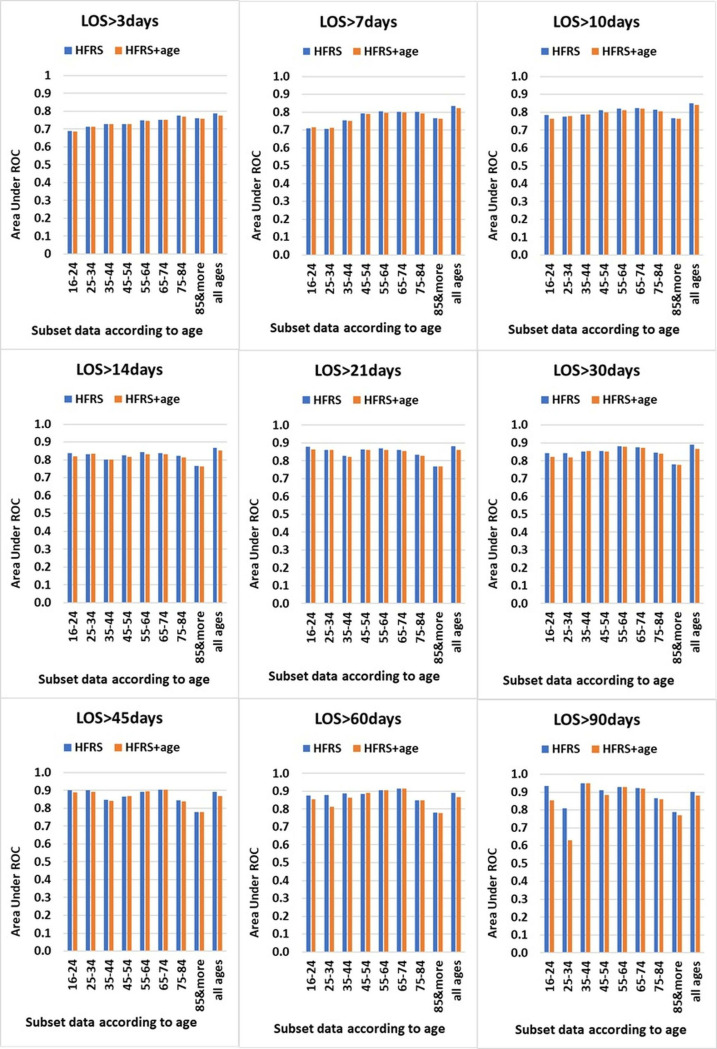
AUROC of HFRS alone and HFRS combined with age according to age groups for 9 LOS.

Those are the results with models using HFRS alone (“crude” HFRS). Fig 3 and S3 Table summarise the models that combined HFRS with age. The ability of HFRS alone had better discrimination than HFRS combined with the age variable. In the “all ages” group, the discrimination (AUROC value) of the HFRS alone model was higher than the HFRS combined with age in all periods of length of stay: AUROCs ranged from 0.788 to 0.885 (at least fair and often good discrimination). The AUROC also increased with longer periods of hospital stay. With the exception of age group 35–44 years, the crude HFRS model had better or the same discrimination than HFRS combined with age in all periods of LOS: AUROCs ranged from 0.700 to 0.932 (at least fair, often good, and sometimes excellent). In the 35–44 age group, the crude HFRS model had better or the same discrimination than HFRS combined with age for some of periods of length of stay.

When additional analyses (Paired t-test and Wilcoxon signed-rank test) were performed, we found that, in the 35–44 years age group, the difference in AUROC values between HFRS alone and HFRS+age was not statistically significant (P > 0.05). In the “all ages” group, the difference between HFRS alone and HFRS+age *was* statistically significant (P < 0.001) and in other age groups was statistically significant at the P < 0.05 level.

The relationship between the Hospital Frailty Risk Score and length of stay across all ages group is presented in Table 2. In both crude HFRS models and age-adjusted models, people with intermediate or high frailty risk have, for all LOS, a higher odds ratio compared with the low frailty risk group. When comparing crude models and adjusted models, the crude odds ratios for intermediate risk and high risk were higher than the adjusted odds ratio in all periods of LOS. In addition, crude models had higher discrimination compared to adjusted models for all periods of LOS.

**Table 2:** Relationship between HFRS and 9 period of LOS for all ages group.

Outcomes	Crude^*^ OR (95% CI)	Adjusted^**^ OR (95% CI)
LOS > 3 days		
Low risk (<5)	1.0	1.0
Intermediate risk (5–15)	5.6 (5.5-5.7)	4.7 (4.6-4.8)
High risk (>15)	13.2 (13.0-13.4)	9.4 (9.2-9.6)
AUROC	0.79	0.78
LOS > 7 days		
Low risk (<5)	1.0	1.0
Intermediate risk (5–15)	7.7 (7.6-7.9)	6.1 (5.9-6.2)
High risk (>15)	19.0 (18.6-19.4)	12.0 (11.8-12.3)
AUROC	0.84	0.82
LOS > 10 days		
Low risk (<5)	1.0	1.0
Intermediate risk (5–15)	8.7 (8.5-8.9)	6.6 (6.5-6.7)
High risk (>15)	21.7 (21.3-22.2)	13.2 (12.9-13.5)
AUROC	0.85	0.83
LOS > 14 days		
Low risk (<5)	1.0	1.0
Intermediate risk (5–15)	9.8 (9.6-10.1)	7.4 (7.3-7.6)
High risk (>15)	24.7 (24.0-25.3)	14.8 (14.3-15.2)
AUROC	0.86	0.84
LOS > 21 days		
Low risk (<5)	1.0	1.0
Intermediate risk (5–15)	11.3 (10.9-11.7)	8.6 (8.3-8.9)
High risk (>15)	28.8 (27.8-29.8)	17.5 (16.9-18.1)
AUROC	0.87	0.85
LOS > 30 days		
Low risk (<5)	1.00	1.00
Intermediate risk (5–15)	12.1 (11.5-12.6)	9.5 (9.1-9.9)
High risk (>15)	31.7 (30.4-33.2)	20.4 (19.5-21.4)
AUROC	0.87	0.85
LOS > 45 days		
Low risk (<5)	1.0	1.0
Intermediate risk (5–15)	13.2 (12.3-14.1)	10.8 (10.1-11.5)
High risk (>15)	34.5 (32.3-36.8)	23.9 (22.3-25.6)
AUROC	0.88	0.85
LOS > 60 days		
Low risk (<5)	1.0	1.0
Intermediate risk (5–15)	14.9 (13.5-16.4)	12.9 (11.7-14.3)
High risk (>15)	35.9 (32.7-39.5)	27.8 (25.1-30.8)
AUROC	0.88	0.85
LOS > 90 days		
Low risk (<5)	1.0	1.0
Intermediate risk (5–15)	18.8 (15.6-22.7)	18.6 (15.4-22.5)
High risk (>15)	43.9 (36.6-53.1)	43.2 (35.5-52.7)
AUROC	0.89	0.86

*Crude model: HFRS alone; ** Adjusted model: HFRS combined with age.

Further analyses were conducted separately for non-elective and elective hospital admissions. This study found that HFRS alone had better discrimination than age alone or HFRS combined with the age; as shown in S4 Table. Also, for each age group, HFRS alone delivered better discrimination than HFRS+age for both non-elective admission (AUROC ranged 0.619–0.922) and elective admission (AUROC ranged 0.637–0.920); as shown in S5 and S6 Tables.

Analyses for data after excluding patients who died in the hospital are presented in S7 Table. We obtained the same study results; HFRS alone had the same or better discrimination than HFRS combined with age. In additional analyses for non-liner models using quadratic logistic regression model and comparing AUROC values from linear regression models with non-linear regression models, we found no significant differences between them; as shown in S8 Table.

## Discussion

In this retrospective cohort study, we explored whether age influences the power of the Hospital Frailty Risk Score (HFRS) to predict a long LOS.

Our study found that the correlation between HFRS and age was weak in all age groups (Table 1), and the proportion of intermediate and high HFRS increases with a longer length of stay in all age groups. At least 52% of patients had intermediate or high risk of frailty in all age groups for LOS in excess of 21 days (Fig 2 and S2 Table). This suggests that calculating the Hospital Frailty Risk Score is useful for all hospital in-patients irrespective of their age. Although HFRS was developed and validated to identify older people at a high risk of poor health outcomes in acute care settings [13], studies of younger patients with specific conditions reported that HFRS remained a significant predictor of adverse health outcomes among them [23–27,30]

Our study found that adding age to the HFRS does not improve its ability to predict length of stay. The crude HFRS model offered at least fair, often good, and sometimes excellent discrimination (AUROCs ranging from 0.700 to 0.932) for all periods of LOS in all age groups, except the 35–44 age group where it was still better for some of the LOS periods, Likely due to random variation rather than a real improvement in predictive performance (Fig 3 and S3 Table). The finding was consistent in both elective and non-elective admitted patients.. The mostly static and otherwise marginal changes AUROC values of HFRS alone models compared to HFRS+age models, indicate that HFRS was a good predictor for LOS and age does not improve its predictive ability.

Our findings are consistent with previous studies, using different frailty assessment tools, which indicated that frailty is good independent predictor for poor health outcomes than age [29,35–37].

This finding is also aligned with observations in several other studies of older people that utilised HFRS [15,17]. A study focused on the construction of the HFRS found the intermediate and high frailty risk groups were more important predictors of long length of stay than any other variables in their analysis, including age for older people [17]. Another study found that crude odds ratios (HFRS alone) were a more important predictor of a long length of stay than adjusted odds ratios (HFRS combined with age, sex, CCI, and socioeconomic status) for older people [15]. A large study in Switzerland of the HFRS in those aged 75 years and over (elective and non-elective admissions) also found that that both intermediate and high frailty risk groups had higher odds of a LOS over 10 days. This was only marginally improved by further adjustments which included age [16].

LOS in hospital increases with increasing age but this has been shown to be associated more with frailty than age [2,4,33–38,43]. This agrees with our findings, which indicate the HFRS is not statistically significantly different among age groups, and patients of any age with frailty tend to have a longer length of stay in the hospital.

Strengths of this study include the large data size used, the inclusion of all adults rather than only older people, and the optimal calculation of the HFRS, which included data from the patient’s current (index) admission along with any previous admissions recorded over a two-year period [17]. We tested the models in a variety of scenarios such as with and without those who had died, and using different approaches to analysis such as non-linear methods and across multiple length of stay thresholds with consistent results.

While this study has demonstrated that age does not affect the ability of HFRS to predict longer LOS, it does have some limitations. This study used data from only one secondary care hospital, which may limit the generalizability of the findings. Replicating this research in varied healthcare environments would be beneficial. Also, we used a wide hospital population, including both elective and non-elective episodes, which may obscure the level of prediction in particular cohorts.

Our findings indicate that the Hospital Frailty Risk Score (HFRS) is not significantly different among age groups, that patients of an any age with frailty tend to have a longer length of stay in hospital and that adding age as a variable does not improve the predictive power of a frailty risk score. The HFRS tool is a frailty risk identification tool based on electronic records and does not capture the full multi-dimensional spectrum of frailty (for example, functional ability). It may not capture all those with frailty, and demonstration of statistically significant findings does not indicate clinical utility. However, there are few frailty identification tools that have focused on all adult ages and developing ways to identify younger groups of in-patients at particular risk is important so that approaches to management can be effectively targeted. The smaller proportion of people in younger age groups who may have frailty means that creating methods that can be applied in very broad range of individuals to identify those at heightened risk may be useful.

Further research could focus on whether these findings are replicated in other settings and on evaluating whether using the HFRS for all hospitalised adults to identify those at risk of long hospital length of stay is helpful to develop pathways, deliver interventions and improve outcomes.

## Conclusion

The Hospital Frailty Risk Score (HFRS) has been validated to identify patients at high risk of adverse outcomes, including long LOS. We found that in this patient population, age does not influence the ability of HFRS to predict long length of stay. This finding demonstrates the utility of HFRS across all ages to predict length of stay.

## Supporting information

S1 Table Characteristics of the study population according to age groups. (DOCX)

S2 Table Relation between Hospital Frailty Risk Score and length of stay. (DOCX)

S3 Table AUROC for HFRS alone and HFRS combined with age for each age groups and length of stay for all admissions. (DOCX)

S4 Table Area Under ROC for 9 periods of long length of stay for all patients, elective patients, and non-elective patients for HFRS alone, age alone and HFRS combined with age. (DOCX)

S5 Table AUROC for HFRS alone and HFRS combined with age for each age groups and length of stay for non-elective admissions. (DOCX)

S6 Table AUROC for HFRS alone and HFRS combined with age for each age groups and length of stay for elective admissions. (DOCX)

S7 Table S(6a-6b): Area Under ROC for 9 periods of long length of stay and 8 age groups for HFRS alone and HFRS combined with age for data after excluded patients who died in hospital. (DOCX)

S8 Table AUROC for HFRS alone and HFRS combined with age for linear and non-linear models, and length of stay. (DOCX)

S1 Fig The top 10 admission speciality. (TIF)
