# Supplementary material for: Age does not improve the predictive ability of the Hospital Frailty Risk Score for length of stay
Source: PLoS One. 2025 Sep 9;20(9):e0330930. doi: 10.1371/journal.pone.0330930 (PMC12419641; doi:10.1371/journal.pone.0330930)
Supplement: S1 Table — (DOCX) [file pone.0330930.s001.docx]

**S1 Table. Characteristics of the study population according to age groups**

|  | **All ages (n=966722)** | **Age groups** |  |  |  |  |  |  |  |
| --- | --- | --- | --- | --- | --- | --- | --- | --- | --- |
|  |  | **16-24 years (n=43109)** | **25-34 years (n=72517)** | **35-44 years (n=75919)** | **45-54 years (n=120971)** | **55-64 years (n=160940)** | **65-74 years (n=204427)** | **75-84 years (n=182234)** | **≥ 85 years (n=106608)** |
| **In-hospital mortality No. (%*)** | 16748 (1.7%) | 16 (0.04%) | 57 (0.08%) | 144 (0.2%) | 464 (0.4%) | 1198 (0.7%) | 2805 (1.4%) | 5106 (2.8%) | 6958 (6.5%) |
| **Type of Admission** |  |  |  |  |  |  |  |  |  |
| Elective admission No. (%*) | 587806 (60.8%) | 15731 (36.5%) | 34326 (47.3%) | 44522 (58.6%) | 81090 (67.0%) | 116869 (72.6%) | 146044 (71.4%) | 109575 (60.1%) | 39649 (37.2%) |
| Non-Elective admission  No. (%*) | 378916 (39.2%) | 27378 (63.5%) | 38188 (52.7%) | 31397 (41.4%) | 39881 (33.0%) | 44071 (27.4%) | 58383 (28.6%) | 72659 (39.9%) | 66959 (62.8%) |
| **Admission Speciality: top 10 No. (%*)** |  |  |  |  |  |  |  |  |  |
| General Medicine (300) | 154180 (16%) | 5268 (12.2%) | 7303 (10.1%) | 8077 (13.3%) | 13236 (10.9%) | 16741 (10.4%) | 25560 (12.5%) | 37798 (20.7%) | 40197 (37.7%) |
| Gastroenterology (301) | 78333 (8.1%) | 1589 (3.7%) | 4033 (5.6%) | 5971 (7.9%) | 11442 (9.5%) | 15669 (9.7%) | 19960 (9.8%) | 15302 (8.4%) | 4367 (4.1%) |
| Medical Oncology (370) | 67451 (7.0%) | 266 (0.6%) | 1422 (2.0%) | 4980 (6.6%) | 11701 (9.7%) | 16576 (10.3%) | 21758 (10.6%) | 9945 (5.5%) | 803 (0.8%) |
| Gynaecology (502) | 50046 (5.2%) | 7631 (17.7%) | 16061 (22.1%) | 10086 (13.3%) | 7243 (6.0%) | 3784 (2.4%) | 2819 (1.4%) | 1824 (1.0%) | 598 (0.6%) |
| Accident & Emergency (180) | 46250 (4.8%) | 5149 (11.9) | 5661 (7.8%) | 5091 (6.7%) | 5776 (4.8%) | 4641 (2.9%) | 4731 (2.3%) | 6971 (3.8%) | 8230 (7.7%) |
| Rheumatology (410) | 43863 (4.5%) | 1659 (3.8%) | 4324 (6.0%) | 4974 (6.6%) | 8651 (7.2%) | 9737 (6.1%) | 8844 (4.3%) | 4711 (2.6%) | 963 (0.9%) |
| Haematology – Clinical (303) | 38348 (4.0%) | 481 (1.1%) | 702 (1.0%) | 1119 (1.5%) | 3174 (2.6%) | 6310 (3.9%) | 12859 (6.3%) | 10101 (5.5%) | 3602 (3.4%) |
| Clinical Oncology (800) | 33121 (3.4%) | 33 (0.1%) | 304 (0.4%) | 1040 (1.4%) | 3964 (3.3%) | 8009 (5.0%) | 12320 (6.0%) | 6761 (3.7%) | 390 (0.6%) |
| Cardiology (320) | 31213 (3.2%) | 225 (0.5%) | 423 (0.6%) | 947 (1.2%) | 3317 (2.7%) | 6367 (4.0%) | 8980 (4.4%) | 7874 (4.3%) | 3107 (2.9%) |
| Trauma & Orthopaedic Surgery (110) | 25658 (3.0%) | 1839 (4.3%) | 2355 (3.2%) | 2225 (2.9%) | 2945 (2.4%) | 3141 (2.0%) | 3668 (1.8%) | 4654 (2.6%) | 4831 (4.5%) |

* Percentage of total sample data
