# Supplementary material for: Age does not improve the predictive ability of the Hospital Frailty Risk Score for length of stay
Source: PLoS One. 2025 Sep 9;20(9):e0330930. doi: 10.1371/journal.pone.0330930 (PMC12419641; doi:10.1371/journal.pone.0330930)
Supplement: S2 Table — (DOCX) [file pone.0330930.s002.docx]

**S2 Table: Relation between Hospital Frailty Risk Score and length of stay**

| **LOS periods** | **HFRS Cat.** | **All ages** | **Age groups** | | | | | | | |
| --- | --- | --- | --- | --- | --- | --- | --- | --- | --- | --- |
|  |  |  | **16-24 years** | **25-34 years** | **35-44 years** | **45-54 years** | **55-64 years** | **65-74 years** | **75-84 years** | **≥ 85 years** |
| **LOS≤3 days** | Low | 84.4% | 93% | 91.9% | 91.2% | 89.2% | 88.5% | 84.7% | 77.3% | 60.6% |
|  | Intermediate | 11.9% | 6.4% | 7.3% | 7.9% | 9.3% | 9.6% | 12.5% | 16.4% | 23.8% |
|  | High | 3.7% | 0.6% | 0.8% | 0.9% | 1.5% | 1.9% | 2.8% | 6.3% | 15.6% |
| **LOS>3 days** | Low | 41.9% | 76.9% | 70.5% | 67.1% | 62.1% | 56.6% | 50.1% | 35.4% | 19.5% |
|  | Intermediate | 33.7% | 18.6% | 24.6% | 26.4% | 28.6% | 30.8% | 33.4% | 36.4% | 37.2% |
|  | High | 24.4% | 4.5% | 4.9% | 6.5% | 9.3% | 12.6% | 16.5% | 28.2% | 43.3% |
| **LOS>7 days** | Low | 30.2% | 65.2% | 60.3% | 56.9% | 49.9% | 45.2% | 38.8% | 26.3% | 15.3% |
|  | Intermediate | 37.8% | 27.3% | 30.5% | 32.9% | 37.2% | 37.5% | 38.7% | 39.2% | 37.4% |
|  | High | 32% | 7.5% | 9.2% | 10.2% | 12.9% | 17.3% | 22.5% | 34.5% | 47.3% |
| **LOS>10 days** | Low | 25.9% | 61.2% | 56.6% | 53.3% | 45.5% | 40.3% | 34.6% | 22.9% | 13.4% |
|  | Intermediate | 38.7% | 29.4% | 32.5% | 35.9% | 39.9% | 39.8% | 40.2% | 39.5% | 37.4% |
|  | High | 35.4% | 9.4% | 10.9% | 10.8% | 14.6% | 19.9% | 25.2% | 37.6% | 49.2% |
| **LOS>14 days** | Low | 22.3% | 53.5% | 50.1% | 48.7% | 41.2% | 36.1% | 30.7% | 19.5% | 11.7% |
|  | Intermediate | 39.5% | 33.3% | 36.6% | 38.5% | 42.3% | 41.7% | 41.5% | 40.7% | 37.2% |
|  | High | 38.2% | 13.2% | 13.3% | 12.8% | 16.5% | 22.2% | 27.8% | 39.8% | 51.1% |
| **LOS>21 days** | Low | 18.7% | 47.6% | 43.5% | 42.5% | 37.2% | 31.8% | 25.9% | 16.3% | 9.8% |
|  | Intermediate | 39.7% | 37.3% | 40.7% | 42.7% | 45.3% | 43.9% | 42.6% | 40.4% | 36.3% |
|  | High | 41.6% | 15.1% | 15.8% | 14.8% | 17.5% | 24.3% | 31.5% | 43.3% | 53.9% |
| **LOS>30 days** | Low | 16.7% | 45.6% | 38.6% | 40.5% | 34.7% | 28.7% | 23.1% | 14.3% | 8.5% |
|  | Intermediate | 39.2% | 41.7% | 42.7% | 41.2% | 46.4% | 44.7% | 43.2% | 39.4% | 34.7% |
|  | High | 44.1% | 12.7% | 18.7% | 18.3% | 18.9% | 26.6% | 33.7% | 46.3% | 56.8% |
| **LOS>45 days** | Low | 15.1% | 31.2% | 30.7% | 42.9% | 29.9% | 22.1% | 20.1% | 13.5% | 7.6% |
|  | Intermediate | 39.3% | 54.2% | 49.1% | 33.8% | 49.2% | 49.2% | 42.1% | 38.9% | 33.7% |
|  | High | 45.6% | 14.6% | 20.2% | 23.3% | 20.9% | 28.7% | 37.8% | 47.6% | 58.7% |
| **LOS>60 days** | Low | 13.9% | 28.5% | 30.1% | 37.3% | 27.2% | 18.9% | 18.7% | 11.3% | 6.7% |
|  | Intermediate | 41.2% | 53.7% | 50.7% | 34.6% | 50.6% | 50.8% | 43.6% | 39.5% | 36.2% |
|  | High | 44.9% | 17.8% | 19.2% | 28.1% | 22.2% | 30.3% | 37.7% | 49.2% | 57.1% |
| **LOS>90 days** | Low | 11.4% | 25.1% | 20.0% | 22.2% | 20.4% | 11.6% | 16.7% | 7.7% | 5.7% |
|  | Intermediate | 42.8% | 56.2% | 56.0% | 40.7% | 59.2% | 53.7% | 43.7% | 39.7% | 33.5% |
|  | High | 45.8% | 18.7% | 24.0% | 37.1% | 20.4% | 34.7% | 39.6% | 52.6% | 60.8% |

**Low:** low frailty risk **(**HFRS < 5); **Intermediate:** intermediate frailty risk (HFRS from 5 to15)**; High:** high frailty risk (HFRS > 15)
