# Supplementary material for: Age does not improve the predictive ability of the Hospital Frailty Risk Score for length of stay
Source: PLoS One. 2025 Sep 9;20(9):e0330930. doi: 10.1371/journal.pone.0330930 (PMC12419641; doi:10.1371/journal.pone.0330930)
Supplement: S3 Table — (DOCX) [file pone.0330930.s003.docx]

**S3 Table: AUROC for HFRS alone and HFRS combined with age For age groups and length of stay**

| **LOS periods** | **model** | **All ages** | **Age groups** | | | | | | | |
| --- | --- | --- | --- | --- | --- | --- | --- | --- | --- | --- |
|  |  |  | **16-24 years** | **25-34 years** | **35-44 years** | **45-54 years** | **55-64 years** | **65-74 years** | **75-84 years** | **≥ 85 years** |
| **LOS>3 days** | **HFRS alone** | 0.788 | 0.700 | 0.714 | 0.729 | 0.729 | 0.748 | 0.753 | 0.775 | 0.761 |
|  | **HFRS+age** | 0.777 | 0.698 | 0.714 | 0.728 | 0.729 | 0.746 | 0.752 | 0.769 | 0.757 |
| **LOS>7 days** | **HFRS alone** | 0.834 | 0.743 | 0.772 | 0.766 | 0.788 | 0.798 | 0.801 | 0.808 | 0.757 |
|  | **HFRS+age** | 0.821 | 0.736 | 0.767 | 0.766 | 0.787 | 0.793 | 0.800 | 0.801 | 0.730 |
| **LOS>10 days** | **HFRS alone** | 0.847 | 0.776 | 0.787 | 0.787 | 0.813 | 0.816 | 0.816 | 0.816 | 0.753 |
|  | **HFRS+age** | 0.824 | 0.771 | 0.775 | 0.787 | 0.810 | 0.811 | 0.815 | 0.808 | 0.750 |
| **LOS>14 days** | **HFRS alone** | 0.858 | 0.783 | 0.806 | 0.807 | 0.838 | 0.83 | 0.831 | 0.825 | 0.754 |
|  | **HFRS+age** | 0.840 | 0.776 | 0.792 | 0.805 | 0.836 | 0.826 | 0.829 | 0.817 | 0.752 |
| **LOS>21 days** | **HFRS alone** | 0.868 | 0.813 | 0.835 | 0.819 | 0.845 | 0.851 | 0.850 | 0.833 | 0.755 |
|  | **HFRS+age** | 0.846 | 0.808 | 0.817 | 0.824 | 0.844 | 0.842 | 0.850 | 0.825 | 0.752 |
| **LOS>30 days** | **HFRS alone** | 0.873 | 0.820 | 0.850 | 0.823 | 0.848 | 0.860 | 0.864 | 0.841 | 0.755 |
|  | **HFRS+age** | 0.847 | 0.821 | 0.826 | 0.819 | 0.848 | 0.858 | 0.861 | 0.835 | 0.754 |
| **LOS>45 days** | **HFRS alone** | 0.879 | 0.886 | 0.851 | 0.797 | 0.890 | 0.884 | 0.872 | 0.842 | 0.756 |
|  | **HFRS+age** | 0.852 | 0.879 | 0.812 | 0.806 | 0.891 | 0.885 | 0.872 | 0.837 | 0.756 |
| **LOS>60 days** | **HFRS alone** | 0.878 | 0.875 | 0.872 | 0.820 | 0.904 | 0.880 | 0.872 | 0.844 | 0.761 |
|  | **HFRS+age** | 0.852 | 0.854 | 0.819 | 0.809 | 0.904 | 0.880 | 0.868 | 0.842 | 0.761 |
| **LOS>90 days** | **HFRS alone** | 0.885 | 0.928 | 0.867 | 0.864 | 0.932 | 0.900 | 0.884 | 0.853 | 0.792 |
|  | **HFRS+age** | 0.864 | 0.834 | 0.846 | 0.911 | 0.932 | 0.900 | 0.884 | 0.853 | 0.792 |
