# Supplementary material for: Age does not improve the predictive ability of the Hospital Frailty Risk Score for length of stay
Source: PLoS One. 2025 Sep 9;20(9):e0330930. doi: 10.1371/journal.pone.0330930 (PMC12419641; doi:10.1371/journal.pone.0330930)
Supplement: S4 Table — (DOCX) [file pone.0330930.s004.docx]

**S4 Table.** **Area Under ROC for 9 periods of long length of stay for all patients, elective patients, and non-elective patients for HFRS alone, age alone and HFRS combined with age**

|  | Length of Stay (LOS) group in day | | | | | | | | |
| --- | --- | --- | --- | --- | --- | --- | --- | --- | --- |
| **All admissions** | **LOS>3** | **LOS>7** | **LOS>10** | **LOS>14** | **LOS>21** | **LOS>30** | **LOS>45** | **LOS>60** | **LOS>90** |
| HFRS alone | 0.788 | 0.834 | 0.847 | 0.858 | 0.868 | 0.873 | 0.879 | 0.878 | 0.885 |
| Age alone | 0.682 | 0.729 | 0.745 | 0.753 | 0.756 | 0.748 | 0.738 | 0.716 | 0.677 |
| HFRS+Age | 0.777 | 0.821 | 0.824 | 0.840 | 0.846 | 0.847 | 0.857 | 0.852 | 0.864 |
| **Non-elective admissions** | **LOS>3** | **LOS>7** | **LOS>10** | **LOS>14** | **LOS>21** | **LOS>30** | **LOS>45** | **LOS>60** | **LOS>90** |
| HFRS alone | 0.725 | 0.757 | 0.767 | 0.775 | 0.784 | 0.790 | 0.790 | 0.788 | 0.790 |
| Age alone | 0.714 | 0.733 | 0.738 | 0.738 | 0.734 | 0.720 | 0.704 | 0.682 | 0.624 |
| HFRS+Age | 0.747 | 0.772 | 0.779 | 0.782 | 0.783 | 0.779 | 0.773 | 0.767 | 0.758 |
| **Elective admissions** | **LOS>3** | **LOS>7** | **LOS>10** | **LOS>14** | **LOS>21** | **LOS>30** | **LOS>45** | **LOS>60** | **LOS>90** |
| HFRS alone | 0.662 | 0.768 | 0.798 | 0.827 | 0.856 | 0.866 | 0.874 | 0.888 | 0.923 |
| Age alone | 0.576 | 0.616 | 0.628 | 0.634 | 0.620 | 0.611 | 0.596 | 0.627 | 0.676 |
| HFRS+Age | 0.649 | 0.739 | 0.764 | 0.784 | 0.805 | 0.823 | 0.830 | 0.859 | 0.913 |
