# Supplementary material for: Age does not improve the predictive ability of the Hospital Frailty Risk Score for length of stay
Source: PLoS One. 2025 Sep 9;20(9):e0330930. doi: 10.1371/journal.pone.0330930 (PMC12419641; doi:10.1371/journal.pone.0330930)
Supplement: S5 Table — (DOCX) [file pone.0330930.s005.docx]

S5 Table. S(5a-5b): Area Under ROC for 9 periods of prediction long length of stay and 8 age groups for HFRS alone and HFRS combined with age for Non-elective admission

S5a Table. Area Under ROC for 9 periods of prediction long length of stay and 8 age groups for HFRS alone for Non-elective admission

| Subset data | **HFRS alone models** | | | | | | | | |
| --- | --- | --- | --- | --- | --- | --- | --- | --- | --- |
|  | **Length of Stay (LOS) group** | | | | | | | | |
|  | **LOS >3 days** | **LOS >7 days** | **LOS >10 days** | **LOS >14 days** | **LOS >21 days** | **LOS >30 days** | **LOS >45 days** | **LOS >60 days** | **LOS >90 days** |
| 16-24 years | 0.652 | 0.725 | 0.745 | 0.772 | 0.833 | 0.848 | 0.903 | 0.922 | 0.913 |
| 25-34 years | 0.674 | 0.737 | 0.760 | 0.792 | 0.838 | 0.837 | 0.884 | 0.902 | 0.895 |
| 35-44 years | 0.677 | 0.709 | 0.739 | 0.760 | 0.787 | 0.795 | 0.798 | 0.828 | 0.889 |
| 45-54 years | 0.659 | 0.708 | 0.724 | 0.745 | 0.770 | 0.792 | 0.805 | 0.838 | 0.827 |
| 55-64 years | 0.672 | 0.709 | 0.724 | 0.740 | 0.754 | 0.774 | 0.800 | 0.813 | 0.823 |
| 65-74 years | 0.654 | 0.685 | 0.702 | 0.715 | 0.736 | 0.753 | 0.760 | 0.769 | 0.767 |
| 75-84 years | 0.653 | 0.679 | 0.687 | 0.693 | 0.704 | 0.715 | 0.717 | 0.719 | 0.744 |
| ≥85 years | 0.619 | 0.629 | 0.632 | 0.638 | 0.640 | 0.645 | 0.638 | 0.626 | 0.650 |

S5b Table. Area Under ROC for 9 periods of prediction long length of stay and 8 age groups for HFRS combined with age for Non-elective admission

| Subset data | **HFRS+age models** | | | | | | | | |
| --- | --- | --- | --- | --- | --- | --- | --- | --- | --- |
|  | **Length of Stay (LOS) group** | | | | | | | | |
|  | **LOS >3 days** | **LOS >7 days** | **LOS >10 days** | **LOS >14 days** | **LOS >21 days** | **LOS >30 days** | **LOS >45 days** | **LOS >60 days** | **LOS >90 days** |
| 16-24 years | 0.650 | 0.722 | 0.743 | 0.767 | 0.783 | 0.826 | 0.902 | 0.890 | 0.878 |
| 25-34 years | 0.671 | 0.726 | 0.741 | 0.782 | 0.821 | 0.808 | 0.834 | 0.879 | 0.859 |
| 35-44 years | 0.670 | 0.704 | 0.735 | 0.755 | 0.784 | 0.801 | 0.790 | 0.822 | 0.843 |
| 45-54 years | 0.654 | 0.701 | 0.713 | 0.734 | 0.773 | 0.777 | 0.802 | 0.834 | 0.814 |
| 55-64 years | 0.667 | 0.699 | 0.711 | 0.722 | 0.738 | 0.760 | 0.788 | 0.809 | 0.818 |
| 65-74 years | 0.654 | 0.684 | 0.699 | 0.713 | 0.736 | 0.751 | 0.758 | 0.768 | 0.765 |
| 75-84 years | 0.652 | 0.676 | 0.683 | 0.689 | 0.700 | 0.711 | 0.707 | 0.711 | 0.734 |
| ≥85 years | 0.618 | 0.629 | 0.632 | 0.636 | 0.640 | 0.643 | 0.631 | 0.618 | 0.658 |
