# Supplementary material for: Age does not improve the predictive ability of the Hospital Frailty Risk Score for length of stay
Source: PLoS One. 2025 Sep 9;20(9):e0330930. doi: 10.1371/journal.pone.0330930 (PMC12419641; doi:10.1371/journal.pone.0330930)
Supplement: S6 Table — (DOCX) [file pone.0330930.s006.docx]

**S6 Table. S(6a-6b): Area Under ROC for 9 periods of prediction long length of stay and 8 age groups for HFRS alone, and HFRS combined with age for elective admission**

S6a Table. Area Under ROC for 9 periods of prediction long length of stay and 8 age groups for HFRS alone for elective admission

| Subset data | **HFRS alone models** | | | | | | | | |
| --- | --- | --- | --- | --- | --- | --- | --- | --- | --- |
|  | **Length of Stay (LOS) group** | | | | | | | | |
|  | **LOS >3 days** | **LOS >7 days** | **LOS >10 days** | **LOS >14 days** | **LOS >21 days** | **LOS >30 days** | **LOS >45 days** | **LOS >60 days** | **LOS >90 days** |
| 16-24 years | 0.729 | 0.787 | 0.788 | 0.804 | 0.850 | 0.842 | 0.888 | 0.852 | 0.852 |
| 25-34 years | 0.707 | 0.812 | 0.839 | 0.865 | 0.895 | 0.888 | 0.799 | 0.801 | 0.801 |
| 35-44 years | 0.662 | 0.762 | 0.790 | 0.832 | 0.861 | 0.912 | 0.797 | 0.900 | 0.900 |
| 45-54 years | 0.642 | 0.749 | 0.792 | 0.784 | 0.841 | 0.854 | 0.838 | 0.901 | 0.901 |
| 55-64 years | 0.654 | 0.764 | 0.773 | 0.800 | 0.812 | 0.810 | 0.905 | 0.842 | 0.842 |
| 65-74 years | 0.637 | 0.756 | 0.789 | 0.816 | 0.839 | 0.865 | 0.842 | 0.931 | 0.931 |
| 75-84 years | 0.645 | 0.737 | 0.769 | 0.810 | 0.851 | 0.865 | 0.920 | 0.852 | 0.852 |
| ≥85 years | 0.707 | 0.773 | 0.805 | 0.829 | 0.867 | 0.877 | 0.888 | 0.763 | 0.763 |

S6b Table. Area Under ROC for 9 periods of prediction long length of stay and 8 age groups for HFRS combined with age for elective admission

| Subset data | **HFRS+age models** | | | | | | | | |
| --- | --- | --- | --- | --- | --- | --- | --- | --- | --- |
|  | **Length of Stay (LOS) group** | | | | | | | | |
|  | **LOS >3 days** | **LOS >7 days** | **LOS >10 days** | **LOS >14 days** | **LOS >21 days** | **LOS >30 days** | **LOS >45 days** | **LOS >60 days** | **LOS >90 days** |
| 16-24 years | 0.718 | 0.787 | 0.786 | 0.781 | 0.778 | 0.789 | 0.846 | 0.679 | 0.679 |
| 25-34 years | 0.710 | 0.811 | 0.819 | 0.842 | 0.889 | 0.748 | 0.799 | 0.763 | 0.763 |
| 35-44 years | 0.657 | 0.762 | 0.788 | 0.797 | 0.836 | 0.909 | 0.761 | 0.888 | 0.888 |
| 45-54 years | 0.645 | 0.739 | 0.772 | 0.757 | 0.792 | 0.829 | 0.844 | 0.897 | 0.897 |
| 55-64 years | 0.637 | 0.743 | 0.761 | 0.781 | 0.811 | 0.801 | 0.900 | 0.837 | 0.837 |
| 65-74 years | 0.632 | 0.754 | 0.785 | 0.814 | 0.824 | 0.855 | 0.843 | 0.930 | 0.930 |
| 75-84 years | 0.637 | 0.729 | 0.762 | 0.795 | 0.844 | 0.864 | 0.900 | 0.679 | 0.679 |
| ≥85 years | 0.705 | 0.764 | 0.786 | 0.809 | 0.861 | 0.868 | 0.846 | 0.801 | 0.801 |
