# Supplementary material for: Age does not improve the predictive ability of the Hospital Frailty Risk Score for length of stay
Source: PLoS One. 2025 Sep 9;20(9):e0330930. doi: 10.1371/journal.pone.0330930 (PMC12419641; doi:10.1371/journal.pone.0330930)
Supplement: S7 Table — (DOCX) [file pone.0330930.s007.docx]

S7 Table. S(7a-7b): Area Under ROC for 9 periods of long length of stay and 8 age groups for HFRS alone and HFRS combined with age for data after excluded patients who died in hospital

**S7a Table. Area Under ROC for 9 periods of prediction long length of stay and 8 age groups for HFRS alone for data after excluded patients who died in hospital**

| Subset data | **HFRS alone models** | | | | | | | | |
| --- | --- | --- | --- | --- | --- | --- | --- | --- | --- |
|  | **Length of Stay (LOS)** | | | | | | | | |
|  | **LOS >3 days** | **LOS >7 days** | **LOS >10 days** | **LOS >14 days** | **LOS >21 days** | **LOS >30 days** | **LOS >45 days** | **LOS >60 days** | **LOS >90 days** |
| 16-24 years | 0.683 | 0.749 | 0.779 | 0.824 | 0.857 | 0.876 | 0.890 | 0.897 | 0.882 |
| 25-34 years | 0.710 | 0.773 | 0.789 | 0.826 | 0.849 | 0.859 | 0.917 | 0.921 | 0.981 |
| 35-44 years | 0.721 | 0.762 | 0.779 | 0.799 | 0.822 | 0.819 | 0.861 | 0.892 | 0.935 |
| 45-54 years | 0.726 | 0.786 | 0.804 | 0.824 | 0.840 | 0.840 | 0.855 | 0.857 | 0.860 |
| 55-64 years | 0.754 | 0.805 | 0.824 | 0.836 | 0.858 | 0.862 | 0.880 | 0.893 | 0.912 |
| 65-74 years | 0.749 | 0.797 | 0.812 | 0.831 | 0.852 | 0.864 | 0.875 | 0.875 | 0.913 |
| 75-84 years | 0.771 | 0.805 | 0.816 | 0.824 | 0.830 | 0.832 | 0.829 | 0.840 | 0.845 |
| ≥85 years | 0.765 | 0.763 | 0.757 | 0.753 | 0.751 | 0.757 | 0.763 | 0.764 | 0.774 |

**S7b Table. Area Under ROC for 9 periods of prediction long length of stay and 8 age groups for HFRS combined with age for data after excluded patients who died in hospital**

| Subset data | **HFRS+age models** | | | | | | | | |
| --- | --- | --- | --- | --- | --- | --- | --- | --- | --- |
|  | **Length of Stay (LOS)** | | | | | | | | |
|  | **LOS >3 days** | **LOS >7 days** | **LOS >10 days** | **LOS >14 days** | **LOS >21 days** | **LOS >30 days** | **LOS >45 days** | **LOS >60 days** | **LOS >90 days** |
| 16-24 years | 0.682 | 0.746 | 0.770 | 0.807 | 0.856 | 0.874 | 0.889 | 0.866 | 0.782 |
| 25-34 years | 0.710 | 0.763 | 0.777 | 0.795 | 0.789 | 0.818 | 0.857 | 0.922 | 0.983 |
| 35-44 years | 0.716 | 0.756 | 0.777 | 0.797 | 0.818 | 0.815 | 0.855 | 0.870 | 0.936 |
| 45-54 years | 0.726 | 0.787 | 0.803 | 0.820 | 0.842 | 0.842 | 0.840 | 0.853 | 0.843 |
| 55-64 years | 0.749 | 0.794 | 0.812 | 0.820 | 0.849 | 0.859 | 0.879 | 0.896 | 0.910 |
| 65-74 years | 0.749 | 0.797 | 0.812 | 0.829 | 0.851 | 0.863 | 0.877 | 0.871 | 0.913 |
| 75-84 years | 0.766 | 0.798 | 0.808 | 0.815 | 0.820 | 0.822 | 0.818 | 0.839 | 0.839 |
| ≥85 years | 0.762 | 0.759 | 0.754 | 0.750 | 0.748 | 0.755 | 0.763 | 0.763 | 0.757 |
