# Supplementary material for: Age does not improve the predictive ability of the Hospital Frailty Risk Score for length of stay
Source: PLoS One. 2025 Sep 9;20(9):e0330930. doi: 10.1371/journal.pone.0330930 (PMC12419641; doi:10.1371/journal.pone.0330930)
Supplement: S8 Table — (DOCX) [file pone.0330930.s008.docx]

**S8 Table: AUROC for HFRS alone, HFRS combined with age for linear and non-linear models,**

**and length of stay**

| **LOS periods** | **HFRS alone models** | **HFRS+age for linear models** | **HFRS+age for non-linear models** |
| --- | --- | --- | --- |
|  | **AUROC** | **AUROC** | **AUROC** |
| **LOS>3 days** | 0.788 | 0.777 | 0.776 |
| **LOS>7 days** | 0.834 | 0.821 | 0.820 |
| **LOS>10 days** | 0.847 | 0.824 | 0.832 |
| **LOS>14 days** | 0.858 | 0.840 | 0.840 |
| **LOS>21 days** | 0.868 | 0.846 | 0.846 |
| **LOS>30 days** | 0.873 | 0.847 | 0.847 |
| **LOS>45 days** | 0.879 | 0.852 | 0.849 |
| **LOS>60 days** | 0.878 | 0.852 | 0.851 |
| **LOS>90 days** | 0.885 | 0.864 | 0.863 |

**Linear models:** logistic regression models; **Non-linear models**: quadratic models
